# Donor- and isolation-related predictive factors of *in vitro* secretory function of cultured human islets

**DOI:** 10.3389/fendo.2024.1345351

**Published:** 2024-02-20

**Authors:** Antoine Buemi, Nizar I. Mourad, Jérôme Ambroise, Delphine Hoton, Arnaud Devresse, Tom Darius, Nada Kanaan, Pierre Gianello, Michel Mourad

**Affiliations:** ^1^Department of Surgery, Surgery and Abdominal Transplantation Division, Université Catholique de Louvain, Cliniques Universitaires Saint-Luc, Brussels, Belgium; ^2^IREC, Pôle de Chirurgie Expérimentale et Transplantation, Institute of Experimental and Clinical Research, Université Catholique de Louvain, Brussels, Belgium; ^3^Institute of Experimental and Clinical Research (IREC), Centre de Technologies Moléculaires Appliquées, Institute of Experimental and Clinical Research, Université Catholique de Louvain, Brussels, Belgium; ^4^Department of Anatomical Pathology, Université Catholique de Louvain, Cliniques Universitaires Saint-Luc, Brussels, Belgium; ^5^Department of Internal Medicine, Nephrology Division, Université Catholique de Louvain, Cliniques Universitaires Saint-Luc, Brussels, Belgium

**Keywords:** islet isolation, islet perifusion, islet function, donor predictive variables, pancreas histology

## Abstract

**Background and aims:**

Human islet preparations designated for research exhibit diverse insulin-secretory profiles. This study aims to assess the impact of donor- and isolation-related factors on *in vitro* islet secretory function.

**Methods:**

A retrospective analysis of 46 isolations from 23 pancreata discarded for clinical transplantation was conducted. *In vitro* islet secretory function tests were performed on Day 1 and Day 7 of culture. Linear mixed-effects models (LMMs) were employed to investigate the relationships between various predictors characterizing the patient and donor characteristics as well as the isolation effectiveness and two functional outcomes including the islet stimulation index (SI) and area under the insulin curve (AUC). Fixed effects were introduced to represent the main effects of each predictor, and backward elimination was utilized to select the most significant fixed effects for the final model. Interaction effects between the timepoint (Day 7 *vs.* Day 1) and the predictors were also evaluated to assess whether predictors were associated with the temporal evolution of SI and AUC. Fold-change (Fc) values associated with each predictor were obtained by exponentiating the corresponding coefficients of the models, which were built on log-transformed outcomes.

**Results:**

Analysis using LMMs revealed that donor body mass index (BMI) (Fc = 0.961, 95% CI = 0.927–0.996, p = 0.05), donor gender (female *vs.* male, Fc = 0.702, 95% CI = 0.524–0.942, p = 0.04), and donor hypertension (Fc = 0.623, 95% CI = 0.466–0.832, p= <0.01) were significantly and independently associated with SI. Moreover, donor gender (Fc = 0.512, 95% CI = 0.302–0.864, p = 0.02), donor cause of death (cerebrovascular accident *vs.* cardiac arrest, Fc = 2.129, 95% CI = 0.915–4.946, p = 0.09; trauma *vs.* cardiac arrest, Fc = 2.129, 95% CI = 1.112–7.106, p = 0.04), pancreas weight (Fc = 1.01, 95% CI = 1.001–1.019, p = 0.03), and islet equivalent (IEQ)/mg (Fc = 1.277, 95% CI = 1.088–1.510, p ≤ 0.01) were significantly and independently associated with AUC. There was no predictor significantly associated with the temporal evolution between Day 1 and Day 7 for both SI and AUC outcomes.

**Conclusion:**

This study identified donor- and isolation-related factors influencing *in vitro* islet secretory function. Further investigations are essential to validate the applicability of these results in clinical practice.

## Introduction

Successful islet transplantation holds the potential to significantly enhance the quality of life for individuals with type 1 diabetes (1, 2). Nonetheless, the scarcity of suitable donor pancreata poses a significant hurdle to the widespread adoption of this therapeutic approach (3).

Donor characteristics and the effectiveness of the islet isolation process are highly related to the success of the procedure (4, 5).

The decision to accept or decline an organ for islet isolation remains challenging, as it relies on various factors predominantly grounded in donor characteristics, coupled with subsequent macroscopic evaluations of the organ (6–10). The capability to reliably reject poor-quality donor pancreata not only improves overall isolation success but also reduces the costs associated with failed isolations (11, 12).

Several donor variables, including donor age, body mass index (BMI), body surface area (BSA), and hemodynamic stability without the use of vasopressors during donor management, have been demonstrated to be associated with higher islet yields, leading to the development of several scoring systems for predicting the suitability of potential pancreatic donors before organ processing (11, 13). However, effective isolation, as assessed by islet yields, may not necessarily translate to good islet graft function after transplantation (14, 15).

Recent investigations have shed light on the significance of non-quantitative indicators in predicting the ultimate goal of any islet transplantation procedure: the normalization of blood sugar levels and relief from diabetic symptoms in transplant recipients (16, 17). Perifusion systems, employed for over 35 years (18–20), challenge islets with glucose and measure dynamic insulin release. They remain the gold standard procedure used by many research laboratories to test islet function in a more physiologically relevant way and attempt to predict islet transplantation outcomes.

However, relatively little attention has been given to donor and preparation characteristics that might influence the insulin-secreting properties of human islets *in vitro* (21, 22). This study aims to retrospectively evaluate, using organs discarded from clinical pancreas and islet transplantation, how features of the preparation and donor attributes impact islet secretory function.

## Research design and methods

From June 2020 to November 2022, a single team at our institution processed 58 consecutive islet isolation procedures from 29 human donor pancreata.

All the organs were divided into two segments, the head–neck portion and the body–tail portion, for use in paired isolation outcomes for other research purposes.

A total of six pancreata were excluded from our analysis for the following reasons: incomplete data (n = 2), technical failure in islet isolation (n = 1), contaminants (n = 1), and islet isolation from partial pancreata (n = 2). The remaining 46 islet isolations from 23 donors were analyzed.

Donor predictor variables used in the analysis were age, gender, body weight, height, BMI, BSA, cold ischemia time (CIT), warm ischemia time (WIT), length of cardiac arrest, cause of death, vasopressor requirement, medical history, and blood test values including peak levels of amylase, lipase, and hemoglobin at the time of procurement. Cause of death was categorized into cerebrovascular accident (CVA), trauma, and cardiac arrest.

Regarding medical history, the following information was collected: alcohol abuse, hypertension (high blood pressure (HBP)), drug abuse, malignancies, smoking, diabetes mellitus, infections, and cardiac arrest.

Islet isolation variables used in the analysis were the islet yield, islet purity, islet size, and pancreas weight.

### Islet isolation

Following the acquisition of informed consent as outlined by the guidelines established by our local medical ethical committee (CEHF, Comité d’éthique hospitalo-facultaire UCL, 2019/07MAI/201), cadaveric pancreata discarded for clinical purposes were obtained from brain-dead donors (DBD) or circulatory-dead donors (DCD) during multiorgan procurement and preserved in Institut Georges Lopez-1 solution.

Upon arrival at our laboratory, each organ was divided into two portions, which were concurrently isolated using a modified automated method (23). The preserved pancreas was perfused through the pancreatic duct with a blend of collagenase NB1 and neutral protease (SERVA Electrophoresis, Heidelberg, Germany), aiming for pancreas distension with minimal leakage.

The distended pancreas underwent dissociation in two separate digestion chambers, a process necessitated by other studies. Islets were subsequently purified through a discontinuous purification method following established protocols (24). The quantification and assessment of islet purity were carried out at the conclusion of the isolation procedure, following established dithizone staining protocols (25). The raw count of islets in each diameter class was determined using an optical graticule. Subsequently, the raw count of islets was converted to the standard number of islet equivalent (IEQ).

Purified islets were cultured in Roswell Park Memorial Institute (RPMI) supplemented with 5 mM glucose, 10% fetal bovine serum (FBS), 100 U/ml penicillin, 100 µg/ml streptomycin, and 1 µg/ml meropenem in 75-cm^2^ culture flasks at 37°C in 5% CO_2_.

### Dynamic glucose-stimulated insulin secretion test

A subset of isolated islets underwent dynamic islet perifusion experiments on Days 1 and 7 of culture, as previously described, to assess *in vitro* functionality (26). The working medium comprised a bicarbonate-buffered solution containing 120 mM NaCl, 4.8 mM KCl, 2.5 mM CaCl_2_, 1.2 mM MgCl_2_, 24 mM NaHCO_3_, 1 mg/ml BSA, and varying glucose concentrations as indicated in the figures. Batches of 1,000–2,000 IEQ were placed in perfusion chambers, covered with 8-μm cellulose filters, and sealed. Test solutions, maintained at 37°C and continuously gassed to stabilize pH ~ 7.2, were pumped at a flow rate of 1 ml/min. Effluent fractions were collected at 2-min intervals and stored for insulin assays using radioimmunoassay (RIA) kits (DiaSource ImmunoAssays, Ottignies-Louvain-la-Neuve, Belgium). At the conclusion of the experiments, islets were recovered, and their insulin content was determined after extraction in acid-ethanol (75% ethanol, 180 mM HCl from Merck, Darmstadt, Germany).

### Statistical analysis

Continuous variables are presented as mean ± SD. Categorical variables are shown as the percentage of the sample.

For the paired analysis, p-values (based on the t-test of least squares means) were reported along with a mean ± SD graph, where appropriate.

p-Values <0.05 were considered to indicate statistical significance. GraphPad Prism (La Jolla, CA, USA) was used for the computation of the descriptive statistics of donor characteristics, islet isolation, islet function, islet size, and histology.

The islet stimulation index (SI) and the area under the insulin curve (AUC) outcomes were modeled using two separate multiple linear mixed-effects models (LMMs), which incorporate all data generated per patient, including both timepoints (Days 1 and 7) and both specimens (head and tail). In each model, fixed effects included various predictors including patients’ characteristics (BMI, age, etc.) as well as isolation characteristics (IEQ/mg). In addition to fixed effects, random intercepts and slopes were introduced to model inter-subject variations. Moreover, interaction effects between the timepoint (i.e., Day 7 *vs.* Day 1) and the predictors were also tested in order to assess whether predictors were associated with the temporal evolution. Backward elimination was used for selecting fixed effects in the final model based on their significance. For both models, a logarithmic transformation was applied to the outcome (i.e., SI and AUC) in order to meet the assumptions of the statistical model (i.e., residuals with normal distribution and homogeneity of variance). Accordingly, coefficients of the corresponding models were exponentiated to obtain the fold changes associated with the predictors in the final models. It is worth noting that multicollinearity issues were mitigated by carefully selecting variables for the model to avoid including highly correlated pairs, such as BMI and BSA.

LMM analyses were performed using the R.4.2.1, as well as the lme4.1.1.33 and lmerTest.3.1.3 packages, while graphical representations of the significant associations between predictors and the outcomes were generated using the ggplot2.3.4.2 package.

## Results

### Donor characteristics, ischemia times, and isolation data

**Tables 1**, **2** present the donor characteristics. The average age of donors was 57.6 years, ranging from 30 to 85 years, with 73.9% being male. The mean BMI and BSA were 26 kg/m^2^ (ranging from 19 kg/m^2^ to 33 kg/m^2^) and 1.96 m^2^ (ranging from 1.63 m^2^ to 2.23 m^2^), respectively. Cerebrovascular accidents were the most frequent cause of death. A majority of donors were DBD (60.8%), and 60.8% of donors received vasopressor therapy during their hospital stay. Smoking and HBP were common. Mean amylase and lipase peak levels were 71 and 38.5 U/L, respectively. The mean lowest hemoglobin level was 11.3 mg/dl. The mean CIT was 940 min, ranging from 704 to 1,765 min, and for DCD donors, the mean WIT was 12.3 min, ranging from 0 to 40 min.

**Table 1:** Donor characteristics, ischemia times, and isolation data from 23 discarded organs for clinical purposes: continuous variables.

Variables	Mean ± SD	Range
Demographics		
Age, (year)	57.6 ± 14.5	30–85
Height (cm)	175 ± 7.2	160–185
Body weight (kg)	79.8 ± 12.6	57–100
BMI (kg/m^2^)	26 ± 3.8	19–33
BSA (m^2^)	1.96 ± 0.17	1.63–2.23
Laboratory results		
Lipase (U/L)	38.5 ± 41.9	7–186
Amylase (U/L)	71 ± 54.4	15–158
Hemoglobin (mg/dl)	11.4 ± 3	6–18.6
Ischemia time		
Cold ischemia time (min)	940 ± 301	704–1,765
Warm ischemia time (min)	12.3 ± 14.6	0–40
Length of cardiac arrest (min)	23.2 ± 13.4	0–40
Isolation data		
Islet yield (IEQ)	67,753 ± 44,513	9,000–141,960
Islet purity (%)	59.8 ± 14	30–90
IEQ/g (IEQ/g)	698.5 ± 470	67–1,549
Pancreas weight (g)	107.8 ± 22.6	84–164

BMI, body mass index; BSA, body surface area; IEQ, islet equivalent; SD, standard deviation.

**Table 2:** Donor characteristics of 23 organs discarded for clinical purposes: categorical variables.

Variables	*N* (%)
Gender	
Male	17 (73.9)
Female	6 (26.1)
Donor organ source	
DBD	14 (60.8)
DCD	9 (39.2)
Cause of death	
CVA	16 (69.5)
Trauma	6 (26)
Cardiac arrest	1 (4.5)
Medical history	
HBP	8 (34.7)
Diabetes mellitus	0 (0)
Smoking	8 (34.7)
Drug abuse	2 (8.7)
Alcohol abuse	4 (17.4)
Infections	5 (21.7)
Malignancies	3 (13)
Cardiac arrest	9 (39.2)
Vasopressor use	14 (60.8)

CVA, cerebrovascular accident; DBD, donation after brain death; DCD, donation after circulatory death; HBP, high blood pressure.

**Figure 1:**
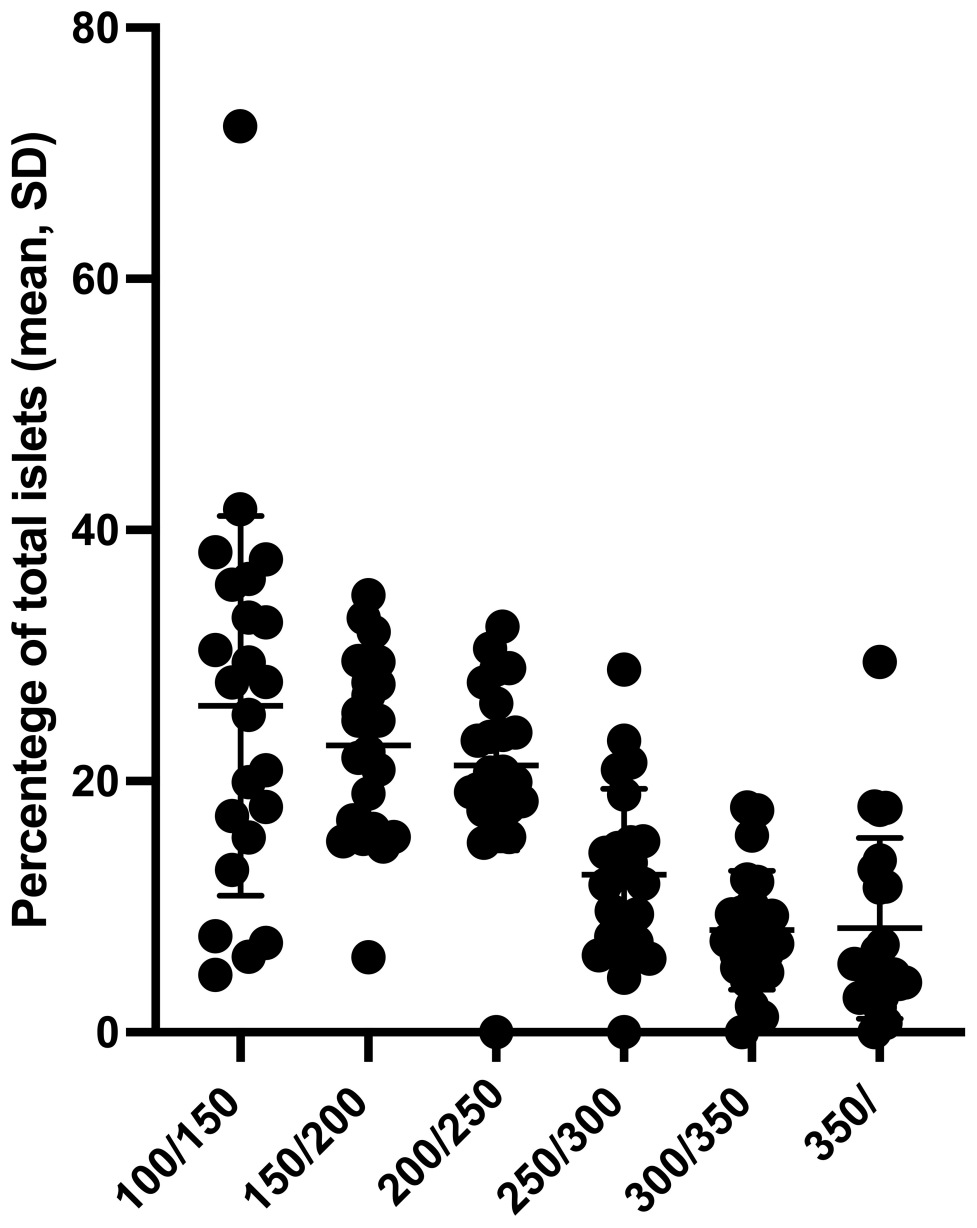
Proportion of islets in different islet size categories of 46 preparations from 23 pancreata discarded for clinical transplantation. SD, standard deviation.

The mean values of IEQ and IEQ/g of the pancreas from these 23 donors were 67,753 ± 44,513 IEQ and 698 ± 470 IEQ/g, respectively. The mean islet purity was 59.8%, ranging from 30% to 90%. **Figure 1** depicts the islet size distribution of the isolation performed in this study.

The proportions of islet average sizes were 25.5% ± 27.5% (100/150 μm), 23.8% ± 8.1% (150/200 μm), 21.9% ± 7.7% (200/250 μm), 13.7% ± 7.2% (250/300 μm), 8.6% ± 4.9% (300/350 μm), and 7% ± 6% (above 350 μm).

### Islet secretory function

**Figure 2:**
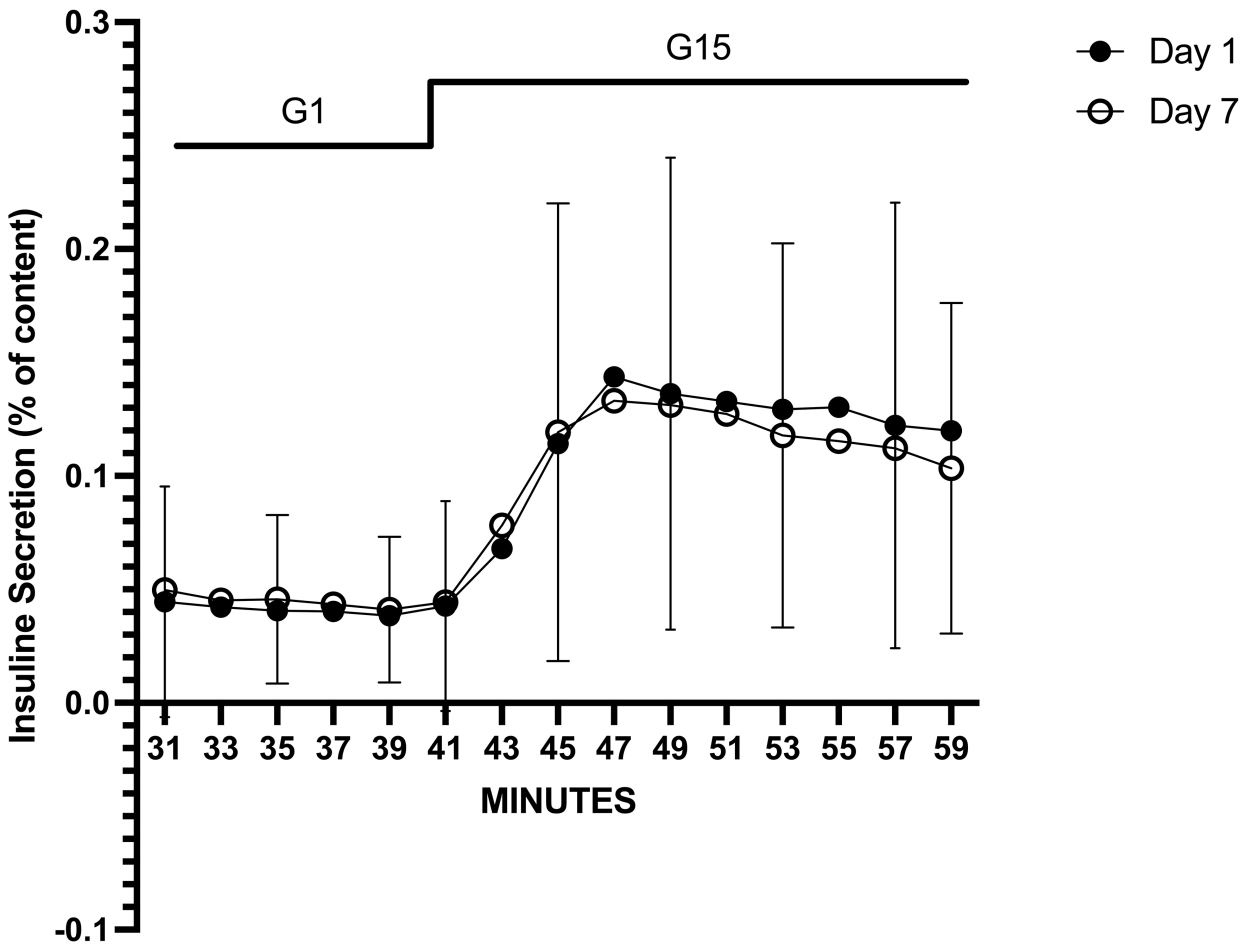
Average glucose-stimulated insulin response from dynamic perifusion experiments on Day 1 and Day 7 of culture in islets obtained from 46 preparations from 23 pancreata discarded for clinical transplantation. AUC was calculated as the sum of insulin secreted during 20 min (from t = 40 to t = 60 min) of stimulation with 15 mM glucose (G15) and expressed as % of total islet insulin content. To calculate a stimulation index (SI), we first calculated the mean rate of insulin secretion (% of content/2 min) during exposure to G1 (basal secretion) and exposure to G15 (stimulated secretion). SI was then calculated as the ratio (or fold-increase) between G15 and G1 (G15/G1). No statistically significant difference was found between Day 1 and Day 7 (p = 0.86). Each timepoint is shown as mean ± SD. AUC, area under the curve.

**Figure 3:**
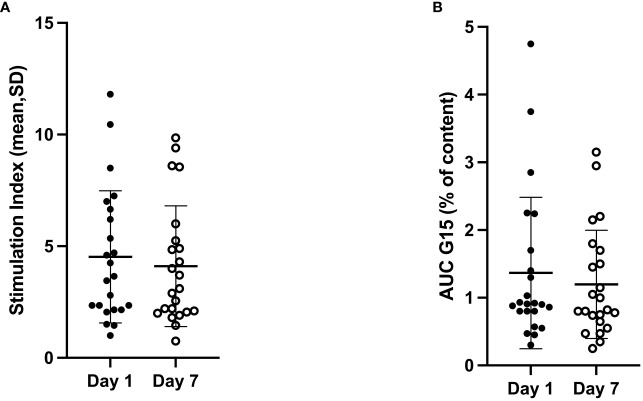
Stimulation index (G15/G1 ratio) **(A)** and AUC of insulin secretion stimulated by 15 mM glucose **(B)** at Day 1 and Day 7 of culture in islets obtained from 46 preparations from 23 pancreata discarded for clinical transplantation. No statistically significant difference was found in terms of both stimulation index (p = 0.58) and AUC of insulin secretion (p = 0.68) between Day 1 and Day 7. AUC, area under the curve.

Dynamic glucose-stimulated insulin secretion tests were performed after 1 day and 7 days of culture (**Figure 2**). In both experiments, islets exhibited a responsive insulin-secretory profile to glucose stimulation, characterized by a sharp, rapid, short-lived increase in insulin secretion (first phase) followed by a lower but constant secretion rate (second phase). The peak stimulation index and the area under the insulin curve were similar on Day 1 and Day 7 (4.36 ± 2.99 *vs.* 4.99 ± 4.60, p = 0.58; 1.41 ± 1.26 *vs.* 1.28 ± 0.98, p = 0.68, respectively) (**Figures 3A, B**).

### Multivariate linear mixed-effect analyses

LMMs followed by predictor selection identified significant positive and negative associations between various predictors describing the isolation effectiveness as well as the donor characteristics and both outcomes of interest (SI and AUC) characterizing the islet secretory function (**Table 3**).

**Table 3:** Positive and negative correlations between the islet secretory function results on Day 1 and Day 7 and the donor and islet isolation variables.

	SI			AUC		
	Fold-change*	95% CI	p	Fold-change*	95% CI	p
Timepoint (Day 7 *vs.* Day 1)	–	–	NS	–	–	NS
Specimen (head *vs.* tail)	–	–	NS	–	–	NS
Age (years)	–	–	NS	–	–	NS
BMI	0.961	0.927–0.996	0.05	–	–	NS
Gender (F *vs.* M)	0.702	0.524–0.942	0.04	0.512	0.302–0.864	0.02
Type donor (DCD *vs.* DBD)	–	–	NS	–	–	–
HBP (yes *vs.* no)	0.623	0.466–0.832	<0.01	–	–	NS
Vasopressors (yes *vs.* no)	–	–	NS	–	–	NS
Cause of death (ref = CA)						
CVA	–	–	NS	2.129	0.915–4.946	0.09
Trauma	–	–	NS	2.811	1.112–7.106	0.04
Pancreas weight (g)	–	–	NS	1.01	1.001–1.019	0.03
IEQ/mg	–	–	NS	1.277	1.088–1.510	<0.01

AUC, area under curve of insulin secretion; BMI, body mass index; CA, cardiac arrest; CVA, cerebrovascular accident; DBD, donation after brain death; DCD, donation after circulatory death; HBP, high blood pressure IEQ, islet equivalent; SI, stimulation index (G15/G1 ratio).

*Coefficient (exponentiated) refers to the impact of donor- and isolation-related variables on the islet secretory function.

NS, Not Significant.

**Figure 4:**
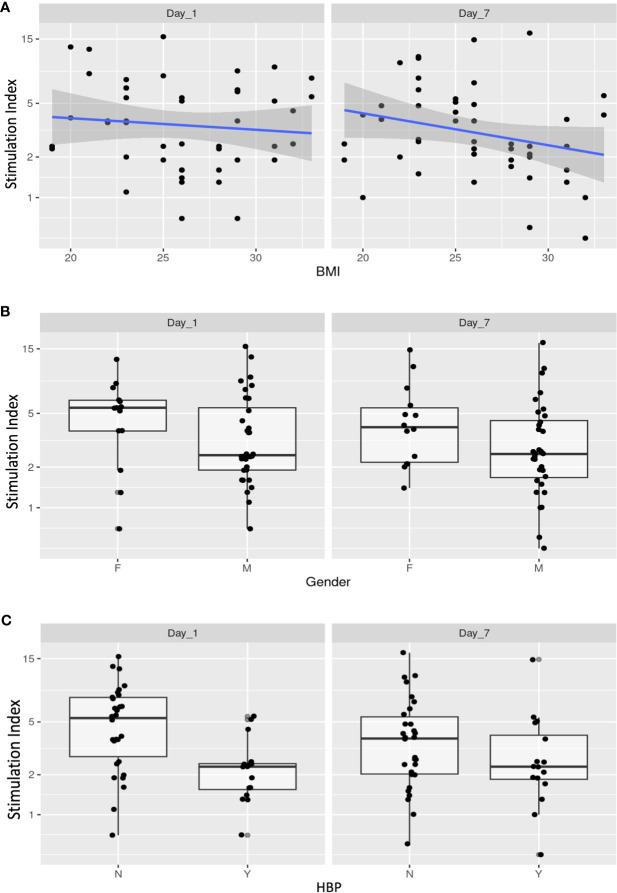
Donor- and isolation-related variables with the stimulation index (G15/G1 ratio) of Day 1 and Day 7 cultured islets. Donor and islet isolation variables displaying significant associations with SI on Day 1 and Day 7 included donor BMI (Fc = 0.961, 95% CI = 0.927–0.996, p = 0.05) **(A)**, donor gender (female *vs.* male, Fc = 0.702, 95% CI = 0.524–0.942, p = 0.04) **(B)**, and donor hypertension (Fc = 0.623, 95% CI = 0.466–0.832, p < 0.01) **(C)**. BMI, body mass index; HBP, high blood pressure; SI, stimulation index.

Predictors displaying significant associations with SI included donor BMI (Fc = 0.961, 95% CI = 0.927–0.996, p = 0.05), donor gender (female *vs.* male, Fc = 0.702, 95% CI = 0.524–0.942, p = 0.04), and donor hypertension (Fc = 0.623, 95% CI = 0.466–0.832, p < 0.01) (**Figure 4**).

**Figure 5:**
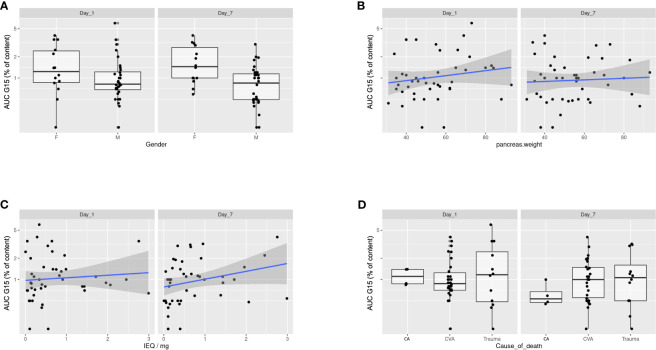
Donor- and isolation-related variables with the AUC of insulin secretion of Day 1 and Day 7 cultured islets. Donor and islet isolation variables related to the AUC of insulin secretion at both Day 1 and Day 7 included donor gender (Fc = 0.512, 95% CI = 0.302–0.864, p = 0.02) **(A)**, pancreas weight (Fc = 1.01, 95% CI = 1.001–1.019, p = 0.03) **(B)**, IEQ/mg (Fc = 1.277, 95% CI = 1.088–1.510, p < 0.01) **(C)**, and donor cause of death (CVA *vs.* CA, Fc = 2.129, 95% CI = 0.915–4.946, p = 0.09; trauma *vs.* CA, Fc = 2.129, 95% CI = 1.112–7.106, p = 0.04) **(D)**. AUC, area under the curve; CA, cardiac arrest; CVA, cerebrovascular accident; IEQ, islet equivalent.

It is noteworthy that because BMI is a continuous variable, the Fc is therefore associated with a one-unit increase in this variable. Variables related to the AUC included donor gender (male *vs.* female, Fc = 0.512, 95% CI = 0.302–0.864, p = 0.02), donor cause of death (cerebrovascular accident *vs.* cardiac arrest, Fc = 2.129, 95% CI = 0.915–4.946, p = 0.09; trauma *vs.* cardiac arrest, Fc = 2.129, 95% CI = 1.112–7.106, p = 0.04), pancreas weight (Fc = 1.01, 95% CI = 1.001–1.019, p = 0.03), and IEQ/mg (Fc = 1.277, 95% CI = 1.088–1.510, p < 0.01) (**Figure 5**).

Furthermore, no significant interaction effects were observed between the timepoint predictor (Day 7 *vs.* Day 1) and any of the other predictors for either SI or AUC, suggesting that no donor- or isolation-related variables could reliably predict the temporal evolution of these outcomes between Day 1 and Day 7.

## Discussion

The present study has identified donor- and isolation-related factors that are significantly positively or negatively associated with the islets’ secretory function after 1 day and 7 days of culture.

We first demonstrated that islet yield expressed as IEQ/g from islet isolation procedures is positively correlated to the islet secretory function expressed by the AUC of the insulin secretion.

Although this observation appears intuitive, it can have encouraging implications in the clinical setting.

Islet yield is the most common indicator in predicting both islet isolation and transplantation outcomes in diabetic patients (3–13).

Clinical studies have generated inconsistent findings concerning the correlation between transplanted IEQ and the likelihood of achieving insulin independence (14, 15). The inaccuracies associated with manual quantification of islet mass, characterized by subjectivity and the potential for error, likely contribute to these inconsistencies. Substantial efforts have been dedicated to overcoming the limitations of standard manual islet mass evaluation through the exploration of computer-assisted and digital-assisted approaches (27–30). Nevertheless, as of now, no precise method has been established.

In contrast, *in vitro* islet function tests are an objective method much less prone to subjective variability, possibly becoming a potentially more accurate indicator than islet quantification.

Nevertheless, since no specific pre-transplant test for evaluating islet quality has demonstrated predictive efficacy for the success of islet transplantation (31), our findings need validation in transplanted islets. This validation process may pave the way for the widespread acceptance of this predictive indicator.

Our study also allowed us to determine several donor characteristics affecting *in vitro* islet secretory function, including the donor BMI, gender, HBP cause of death, and pancreas weight.

Numerous studies have highlighted a positive correlation between donor BMI and successful islet yield outcomes (13, 32, 33). Conversely, donors with a low BMI (<21 kg/m^2^) often yield less satisfactory results in terms of islet production (34). However, as previously discussed, the critical focus lies in evaluating the functionality of islets obtained from obese donors. In a specific study, islets isolated from donors with a BMI exceeding 30 kg/m^2^ displayed nearly identical viability (33). This was determined through *in vitro* assessments, including insulin response to glucose in static incubation assays, as well as *in vivo* evaluations, such as the time required to reverse chemical diabetes in a nude mouse bioassay.

Remarkably, our research indicates that although there is no statistically significant correlation between BMI and the AUC of insulin secretion, SI increases with the gradual reduction of BMI. These findings, prompting further exploration, may be influenced by confounding factors. On the one hand, the origin of donor organs—rejected for clinical use—often entails higher overall risk factors than commonly utilized organs. On the other hand, longer ischemia times, exceeding those typically accepted by the scientific community, could also contribute to these outcomes.

Furthermore, our study provides valuable insights by establishing a positive correlation between organ weight and the functional activity of isolated islets. Numerous studies have consistently shown a positive association between pancreas weight and both pre-purification and post-purification islet yields (13, 35). However, it is crucial to note that this parameter remains indeterminable prior to organ procurement, prompting a focus on identifying metrics reliably correlated with pancreas weight. As a result, common practice has adopted body weight and body surface area as crucial metrics for organ selection and predicting outcomes in islet isolation (35). The crucial impact of organ weight on islet function underscores the importance of accurately predicting this parameter.

Recent evidence indicates that recipients of islets from at least one female donor experienced prolonged graft survival in comparison to recipients exclusively receiving male donor islets (36). This observation is believed to be associated with the cell-intrinsic properties of female islets (37). A study reported that the higher β-cell content in transplanted islets from female donors led to significant improvements in recipient outcomes compared to islets transplanted from male donors (38).

Our data also show that male donor is negatively related to the *in vitro* islet secretory function.

Our study additionally reveals that donor hypertension (HBP) is linked to decreased *in vitro* functional performance of isolated islets in terms of AUC. Drawing insights from kidney transplant research, the authors posit that the adverse impact of HBP on islet function might stem from vascular damage, resulting in a compromised organ blood supply (39). Furthermore, a recent study reported that donors with a history of HBP exhibited increased pancreatic lipid content and islet lipid content, irrespective of gender. This association was correlated with poorer islet performance after isolation (40).

Finally, our findings substantiate a noteworthy negative correlation between cardiac arrest as a cause of death and islet function when contrasted with alternative causes like trauma or cerebrovascular accidents.

This observed correlation could be closely tied to the varying durations of low or absent blood flow during the phases of donor resuscitation. The prolonged periods of diminished perfusion or complete cessation of blood flow during these critical stages exert a detrimental influence on pancreas perfusion, thereby contributing to the development of ischemia–reperfusion injuries (41, 42).

This study has certain limitations, primarily stemming from the relatively low number of cases and the presence of numerous potential confounding variables, mainly associated with the nature of the pancreas discarded for clinical purposes and the extended ischemia times. Additionally, *in vivo* function tests were not conducted in this study.

## Conclusions

In conclusion, this study identified donor- and isolation-related factors that influence *in vitro* islet secretory function. Further studies are essential to evaluate the reliability of these results in clinical practice.

## Data availability statement

The raw data supporting the conclusions of this article will be made available by the authors, without undue reservation.

## Ethics statement

The studies involving humans were approved by Cliniques Universitaires Saint Luc. The studies were conducted in accordance with the local legislation and institutional requirements. The human samples used in this study were acquired from primarily isolated as part of your previous study for which ethical approval was obtained. Written informed consent for participation was not required from the participants or the participants’ legal guardians/next of kin in accordance with the national legislation and institutional requirements.

## Author contributions

AB: Conceptualization, Investigation, Writing – original draft, Writing – review & editing. NM: Investigation, Methodology, Validation, Visualization, Writing – review & editing. JA: Data curation, Methodology, Validation, Writing – review & editing. DH: Methodology, Writing – review & editing. AD: Validation, Visualization, Writing – review & editing. TD: Visualization, Writing – review & editing. NK: Visualization, Writing – review & editing. PG: Visualization, Writing – review & editing. MM: Validation, Writing – review & editing.
